# Regulation Between HSF1 Isoforms and HSPs Contributes to the Variation in Thermal Tolerance Between Two Oyster Congeners

**DOI:** 10.3389/fgene.2020.581725

**Published:** 2020-10-27

**Authors:** Youli Liu, Li Li, Haigang Qi, Huayong Que, Wei Wang, Guofan Zhang

**Affiliations:** ^1^Key Laboratory of Experimental Marine Biology, Institute of Oceanology, Chinese Academy of Sciences, Qingdao, China; ^2^Laboratory for Marine Biology and Biotechnology, Qingdao National Laboratory for Marine Science and Technology, Qingdao, China; ^3^Laboratory for Marine Fisheries Science and Food Production Processes, Qingdao National Laboratory for Marine Science and Technology, Qingdao, China; ^4^Center for Ocean Mega-Science, Chinese Academy of Sciences, Qingdao, China; ^5^National and Local Joint Engineering Laboratory of Ecological Mariculture, Qingdao, China

**Keywords:** thermal stress, HSF1, HSP, regulatory relationship, oyster, comparison

## Abstract

Heat shock transcription factor 1 (HSF1) plays an important role in regulating heat shock, which can activate heat shock proteins (HSPs). HSPs can protect organisms from thermal stress. Oysters in the intertidal zone can tolerate thermal stress. The Pacific oyster (*Crassostrea gigas gigas*) and Fujian oyster (*C. gigas angulata*)—allopatric subspecies with distinct thermal tolerances—make good study specimens for analyzing and comparing thermal stress regulation. We cloned and compared HSF1 isoforms, which is highly expressed under heat shock conditions in the two subspecies. The results revealed that two isoforms (HSF1a and HSF1d) respond to heat shock in both Pacific and Fujian oysters, and different heat shock conditions led to various combinations of isoforms. Subcellular localization showed that isoforms gathered in the nucleus when exposed to heat shock. The co-immunoprecipitation revealed that HSF1d can be a dimer. In addition, we selected *HSP*s that are expressed under the heat shock response, according to the RNA-seq and proteomic analyses. For the HSPs, we analyzed the coding part and the promoter sequences. The result showed that the domains of HSPs are conserved in two subspecies, but the promoters are significantly different. The Dual-Luciferase assay showed that the induced expression isoform HSF1d had the highest activity in *C. gigas gigas*, while the constitutively-expressed HSF1a was most active in *C. gigas angulata*. In addition, variation in the level of *HSP* promoters appeared to be correlated with gene expression. We argue that this gene is regulated based on the different expression levels between the two subspecies’ responses to heat shock. In summary, various stress conditions can yield different HSF1 isoforms and respond to heat shock in both oyster subspecies. Differences in how the isoforms and promoter are activated may contribute to their differential expressions. Overall, the results comparing *C. gigas gigas* and *C. gigas angulata* suggest that these isoforms have a regulatory relationship under heat shock, providing valuable information on the thermal tolerance mechanism in these commercially important oyster species.

## Introduction

Temperature can affect the health (Zhang et al., 2019) and distribution (Viña, 2002; Pinsky et al., 2013) of organisms. Organisms in the intertidal zone may suffer increasingly more stress under global warming (Mcarley et al., 2017). Oysters are known to be resilient to thermal stress and possess expansive heat shock protein (*HSP*) family members. However, large-scale oyster deaths have been documented in recent summers all over the world (Samain and McCombie, 2008; Shiel et al., 2018), and these were thought to be related to high temperature stress (Ugalde et al., 2018; Zhang et al., 2019). Thus, analyzing the mechanisms by which oysters respond to high temperature stress is helpful for understanding these summer deaths. The Pacific oyster (*Crassostrea gigas gigas*) and Fujian oyster (*C. gigas angulata*) are important economic subspecies in northern and southern China, respectively (Wang et al., 2008, 2010). Their distribution was thought to be related to the annual temperature range, and recent research has shown the Pacific and Fujian oysters to be capable of different thermal tolerances (Li et al., 2017; Ghaffari et al., 2019; Li et al., 2019). The two congeners were shown to have significant levels of enzymatic activity, respiration rate, and induced-gene change level in response to heat shock (Li et al., 2017). These previous studies argue that the Fujian oyster is better adapted to high temperature environments than is the Pacific oyster. In addition, it was revealed that the two congeners have significantly different temperature tolerances and temperature-related physiological mechanisms (Ghaffari et al., 2019). The reason for these differences in thermal tolerance and *HSP* expression levels, however, remains unclear.

Heat shock transcription factor (HSF) 1 is a regulatory factor that is essential for regulating the response to heat shock. There are multiple HSFs in plants and animals, but only one in yeast and *Drosophila* (Liu et al., 1997; Scharf et al., 2012; Mahat et al., 2016; Wang et al., 2018). The studies showed that a variety of HSFs can be more efficient than just one. Some studies revealed that eight subtypes of HSF1 in the Pacific oyster respond to various stress conditions (Kawabe and Yokoyama, 2011), but HSFs are rarely studied in Fujian oysters. It was reported that the induced *HSP* is mainly regulated by HSF1 under heat shock (Pirkkala et al., 2001). A previous study showed that HSF1 regulated HSPs by binding to the heat shock elements (HSEs, repeat sequence 5′-nGAAn-3′ or 5′-nTTCn-3′) in the promoter region (Xiao et al., 1991). Some studies revealed that there is a regulatory relationship between HSF1 and HSP in the Pacific oyster (Kawabe and Yokoyama, 2011; Liu et al., 2019, 2020). HSPs are important for protecting an organism from various stresses (Chen et al., 2011). Under heat shock stress, an organism’s HSPs (especially the *HSP70* genes) can be up-regulated significantly (Piano et al., 2002; Meistertzheim et al., 2007; Lim et al., 2016). Thermal tolerance was found to be related to the expression of HSP family members in the Pacific oyster (Hamdoun et al., 2003). However, the correlation between the regulatory relationship and gene expression level variation remains unclear.

A mutation in promoter sequences can affect how transcripts respond to stimuli (Hornung et al., 2012; Carey et al., 2013). In addition, mutations would affect the transcripts more in *cis*-regulated promoters (Metzger et al., 2016). Some studies revealed that mutations in the promoter of yeast metabolic genes change gene expression related to organisms’ abilities to survive and adapt in a specific environment (Duveau et al., 2017). In some species, polymorphisms in *HSP70* genes lead to differential gene expression, which affects the organism’s thermotolerance (Valenzuela-Castillo et al., 2015). There were found to be differences in the HSE types of *HSP70* that contribute to changes in expression level; these in turn led to differential thermotolerance between two congener endemic amphipod species (Bedulina et al., 2013). In copepods, variation in these heat shock-related binding sites is related to the population’s thermal adaptation, and north and south populations were found to have different induced expressions of HSPs (Tangwancharoen et al., 2018). Differences in HSP expression patterns exist in the Pacific oyster and Fujian oyster, but their relationships to thermotolerance are still unclear.

In the present study, we analyzed which isoforms were expressed in response to heat shock. Then, we compared the coding sequences and promoter sequences of candidate *HSP* genes enriched by transcriptomes, chromatin immunoprecipitation, and the results of previous studies. Finally, we analyzed the regulatory relationship of the chosen *HSP* genes.

## Materials and Methods

### Oysters

Wild samples of the two subspecies, *C. gigas gigas* and *C. gigas angulata*, were collected from the Yellow Sea (36°21′N, Qingdao, Shandong Province, China) and the East China Sea (24°33′N, Xiamen, Fujian Province, China), respectively. The oysters were cleaned and acclimated in an aquarium with aerated and sand-filtered seawater for 1 week. *Spirulina* powder was added as a food source and the seawater was changed daily (10–12°C). *C. gigas gigas* and *C. gigas angulata* had a height of 49.62 ± 1.04 and 50.91 ± 1.05 mm (mean ± SEM), respectively.

### Heat Shock Treatment

After the acclimation period, oysters were put into water, which was temporarily increased to a specific temperature (22, 29, 36, 40, or 43°C) and maintained for 1 h. The temperature was controlled by a water bath with a temperature control system. The gills of the oysters were sampled and placed into liquid nitrogen, then stored in a −80°C refrigerator until the next experiment.

### RNA Preparation and Reverse Transcription

After sampling, total RNA was prepared using a TIANGEN RNAprep Pure Tissue Kit (DP431; TIANGEN BIOTECH, Beijing, China) following standard protocols. The quality and quantity of prepared RNA were assessed through 1.5% agarose gel electrophoresis and a NanoDrop 2000c UV-Vis Spectrophotometer (Thermo Fisher Scientific, United States). Each RNA sample was reversed by a complementary DNA (cDNA) synthesis kit (RR047A; Takara, Japan) to detect gene expression.

### Quantitative RT-PCR

To analyze the gene expression patterns under different temperature stresses, we performed q-RT-PCR using the gills sampled from the different temperature heat shocks and primers from a previous study (Kawabe and Yokoyama, 2011). q-RT-PCR was performed in the ABI 7500 Fast Real-Time PCR System (Applied Biosystems, United States) using a SYBR Green real-time PCR mix (RR420A; Takara). Each 20 μl reaction mix included the following: 10 μl of 2SYBR Premix Ex Taq, 0.4 μl each of the forward and reverse primers, 0.4 μl 50ROX Reference DyeII, 2 μl of diluted cDNA, and 6.8 μl of DEPC-treated water. The reaction was performed in 96-well optical plates (Applied Biosystems, United States) under the following conditions: 30 s at 95°C, 3 s at 95°C for 40 cycles, and 30 s at 60°C. The results were analyzed with the 2^–Δ^ ^Δ^ ^*ct*^ method (Livak and Schmittgen, 2001).

### DNA Preparation, Gene Cloning, and Sequence Analysis

DNA was extracted from the samples treated with heat stress using the TIANamp Marine Animals DNA Kit (DP324; TIANGEN), and then stored at −80°C until DNA amplification. Given the various isoforms of *C. gigas gigas HSF-1* (Kawabe and Yokoyama, 2011), it is important to identify which isoforms play important roles under heat shock. The coding sequencing primers of *HSF-1* and *HSP 70* are in Supplementary Table 1. The primers for HSP gene promoter amplification were designed using the whole genome sequence of the Pacific oyster (Zhang et al., 2012) and are presented in Table 1. The PCR was conducted with LAtaq enzyme (RR900A; Takara) using the following program: 35 cycles at 94°C for 30 s, 55°C for 30 s, and 72°C for 2 min followed by an extension at 72°C for 10 min. Then, PCR products were purified (Sangon Biotech, China) and cloned in a pEASY-T1 cloning vector (TransGen Biotech, China). The recombinant vector was transformed into Trans1-T1 competent cell (TransGen) and sequenced by a sequencing company (Personalbio, China). The accession numbers of A-HSF1a, A-HSF1d and the five HSP genes in GenBank are MT737786–MT737796. Then, the sequences were analyzed using bio editing software^1^ and the multiple sequence alignment of clustal omega^2^. Gene functional domains were predicted by simple modular architecture research tool^3^ and national center for biotechnology information (NCBI) conserved domains^4^. We calculated the molecular weight of proteins on the website^5^ (in Chinese). The similarities of candidate HSP genes in the two subspecies were calculated by global alignment in NCBI^6^.

**TABLE 1:** Primers used for HSP promoter cloning.

Primer name	Sequences
10006977-1F	GTTTACAATCTCTTCGTTCTTTCTA
10006977-1R	GATGGAAATGCTTTCTATAGCATAT
10002375-1F	ACAATAAACATTATATAGCCTATAACTATC
10002375-1R	TCGCCATGTTTGTCGATTTGTGAAG
10008834-1F	AGATGTTTACTATGATCACATATATCAG
10008834-1R	GTTGGAGAATTCTGGGATATG
10002594-1F	TGCTTGTTTGTAAACACTAAAGTGAAAG
10002594-1R	ATTGTTAAAACCTGTCACTGCCTT

### Plasmid Construction

The plasmids were constructed via homologous clone using a blunt-end amplification enzyme (Vazyme, Nanjing, China). The primers used for plasmid construction are listed in Table 2. For fragments cloning, following the program: 98°C for 2 min, 30 cycles at 98°C for 10 s, 60°C for 15 s, and 72°C 15 s/Kb, followed by an extension at 72°C for 5 min. After cloning and sequencing, the plasmids were extracted using an endotoxin-free plasmid extraction kit (Tiangen).

**TABLE 2:** Primers used for plasmid construction.

Primer name	Sequences
HSF-EGFP-F	CTCAAGCTTCGAATTCTATGGGTTCAAACCCTGTACCAGCG
HSF-EGFP-R	GTCGACTGCAGAATTCGCAGGTCGTCTGCGGAGATCTGG
HSF-Flag-F	GCTTCTGCAGGAATTCATGGGTTCAAACCCTGTACCAGCG
HSF-Flag-R	CGACGATATCGAATTCTCACAGGTCGTCTGCGGAGATCTGG
HSF-pcDNA3.1-F	GTGGCGGCCGCTCGAGATGGGTTCAAACCCTGTACCAGCG
HSF-pcDNA3.1-R	GCCCTCTAGACTCGAGCAGGTCGTCTGCGGAGATCTGG
10006977-pGL3-basic-1F	CTAGCCCGGGCTCGAGGTTTACAATCTCTTCGTTCTTTCTA
10006977-pGL3-basic-1R	GATCGCAGATCTCGAGGATGGAAATGCTTTCTATAGCATAT
10002375-pGL3-basic-1F	CTAGCCCGGGCTCGAGACAATAAACATTATATAGCCTATAACTATC
10002375-pGL3-basic-1R	GATCGCAGATCTCGAGTCGCCATGTTTGTCGATTTGTGAAG
10008834-pGL3-basic-1F	CTAGCCCGGGCTCGAGAGATGTTTACTATGATCACATATATCAG
10008834-pGL3-basic-1R	GATCGCAGATCTCGAGGTTGGAGAATTCTGGGATATG
10002594-pGL3-basic-1F	CTAGCCCGGGCTCGAGTGCTTGTTTGTAAACACTAAAGTGAAAG
10002594-pGL3-basic-1R	GATCGCAGATCTCGAGATTGTTAAAACCTGTCACTGCCTT

### Subcellular Localization of HSF-1 Isoforms

To identify the distribution of HSF1a and HSF1d in normal situation and under heat shock, we displayed a subcellular localization assay with tags of enhanced green fluorescent protein (EGFP). pEGFP-N1 carrying distinct gene fragments (HSF1a and HSF1d) were transfected into HeLa cells. HeLa cells were cultured at 37°C after transfected. All plates were separated to two groups, one of them was put in 37°C (normal) while another was in 42°C (heat shock) for 30 min. Cells were rinsed once with PBS 24 h later and then stained with Hoechst 33342 (Invitrogen) for 18 min at 37°C. Subsequently, the cells were washed twice with PBS, stained with Alexa Fluor 594 (Life Technologies, Carlsbad, CA, United States) for 15 min at 37°C, washed three times with PBS, and then cultured in modified RPMI-1640 medium without fetal bovine serum (FBS). The visualization of protein subcellular localization was performed by confocal microscopy (Carl Zeiss, Oberkochen, Germany).

### Co-immunoprecipitation Assay (Co-IP)

Co-immunoprecipitation is a method which can be used to detect the interaction between known proteins using the specificity of antigen and antibody. In the experiment, HEK293T cells transfected with plasmids expressing the FLAG and EGFP tags were harvested at 30 h post transfection with cell lysis buffer (Beyotime). Input samples were prepared from the cell lysate. The remaining lysate was mixed with anti-FLAG M2 magnetic beads (Sigma-Aldrich) and shaken gently on a roller shaker for 1–2 h. Subsequently, the magnetic beads were washed three times with cell lysis buffer and incubated with 2 × SDS-PAGE loading buffer (TaKaRa) for 8 min at 100°C to elute the bound protein. Then, the beads were removed and immunoprecipitated proteins were analyzed using western blotting. The FLAG and EGFP antibodies were used to detect the expression of the target protein via western blotting assay with input samples. The samples incubated with the anti-FLAG M2 magnetic beads were used for detecting whether the two labeled proteins can bind by western blotting with the EGFP antibody.

### Dual Luciferase Reporter Assay for Transcriptional Analysis

Human embryonic kidney (HEK) 293T cells (ATCC, Manassas, United States) were cultured in Dulbecco’s modified Eagle’s medium (DMEM) supplemented with 10% FBS and antibiotics (100 U/ml penicillin and 100 U/ml streptomycin) at 37°C and 5% CO_2_ in an incubator. The intended plasmid DNAs were transfected into the cells using Opti-MEM and Lipofectamine 3000 (Life Technologies, Carlsbad, CA, United States), following the manufacturer’s protocol. To determine the transcriptional activity of HSF-1a and HSF-1d of the two subspecies, each pcDNA-HSF1a, pcDNA-HSF1d, and pcDNA3.1 plasmid was transfected into the HEK293 cells. Additionally, pGL3-basic carrying HSPs’ promoter fragments and pRL-TK (a Renilla luciferase reporter plasmid) were co-transfected as the sample and control, respectively, to determine the transfection efficiency. The control group contained pRL-TK, pcDNA3.1 plasmid, and pGL3-basic carrying HSPs’ promoter fragments, while the sample group contained pRL-TK, pcDNA3.1- HSF1a, or pcDNA3.1- HSF1d plasmid and pGL3-basic carrying HSPs’ promoter fragments. Luciferase activity was measured by a dual-luciferase reporter assay system (Promega) in a Varioskan Flash multimode reader (Thermo Scientific, Waltham, MA, United States) 24 h after transfection, following the manufacturer’s instructions. The results were reported as fold increases by comparing them to the control group. The Dual luciferase reporter assay can be used to calculate the regulation level between the transcript factor and the candidate genes (Wang et al., 2016). The number of ratio represents the activation level of the candidate gene compared with the control group.

### Statistical Analyses

Gene expression levels were calculated by the 2^–ΔΔ*ct*^ method (Livak and Schmittgen, 2001). Student’s *t*-test was used to evaluate the gene expression levels or regulation activity of HSF1 isoforms.

## Results

### Characteristics of HSF1 Isoforms

**FIGURE 1:**
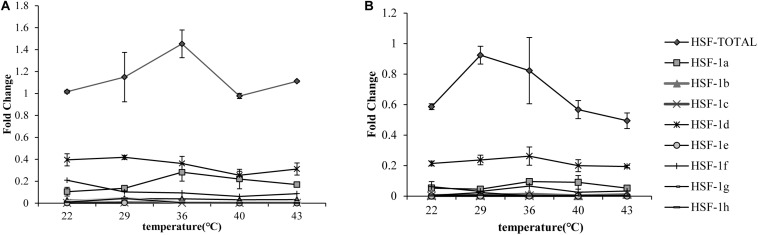
qRT-PCR showing the expression patterns of total HSF1 and eight isoforms of HSF1 in two congener species. **(A)** The expression patterns of HSF1 isoforms in *C. gigas gigas* (*n* = 5). *X*-axis: the fold change in expression levels at 12°C. *Y*-axis: 22, 29, 36, 40, 43 represent different thermal stress conditions. Different lines represent the expression level at each temperature. Error bar denotes the standard error of the mean. **(B)** The expression patterns of HSF1 isoforms in *C. gigas angulata* (*n* = 5). The tags to the right represent each gene.

The qRT-PCR results showed that the total HSF1 expression level was highest at 36°C in the Pacific oyster (Figure 1A) and 29°C (Figure 1B) in the Fujian oyster (values in Supplementary Table 2). The expression levels of HSF1 in the Pacific oyster were higher than Fujian oyster under same treatment. Though differences in the expression level of HSF1 isoforms between treatments were not significant (*P* > 0.05), the results can provide information regarding multiple combinations of isoforms in response to short time heat shock. HSF1a and HSF1d expression level were the highest among the other isoforms, and may play an important role in regulating thermal stress in both Pacific and Fujian oysters according to the expression patterns of various isoforms.

**FIGURE 2:**
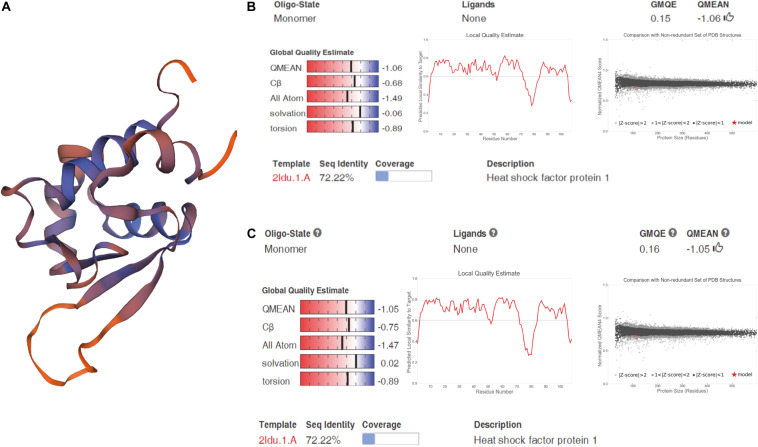
Spatial structure of A-HSF1a and A-HSF1d. **(A)** A protein model of A-HSF1a and A-HSF1d. The human HSF1 protein was matched to the isoforms by SWISS-MODEL. **(B)** Model matching results of A-HSF1a. “GMQE” refers to the Global Model Quality Estimation. Higher numbers indicate higher reliability. “QMEAN” represents the quality estimates, both of the entire structure and per residue. The close the value is to zero, the better the model expect the results from experimental structures of similar size. The close the values of “Cβ,” “All Atom,” “Solvation,” and “torsion” are to 0, the better the fit to the model. “Local Quality Estimate” indicates the expected similarity of each residue (*X*-axis) of the model to the model structure (*Y*-axis). Generally, a value lower than 0.6 is considered a low quality match (the line represents 0.6). The “Comparison” result indicates that the points located in the black zone are a better fit. The gray zone represents a poorer fit. The red star represents the model. The location of the star indicates the fitness of the model. **(C)** Model matching results of A-HSF1d.

We cloned and compared HSF1a and HSF1d isoforms of the Fujian oyster and named them A-HSF1a and A-HSF1d, respectively (Figure 2). We statistically analyzed the differential amino acids in comparison with the Pacific oyster in HSF1a and HSF1d, respectively (the statistics are listed in Table 3). Eight amino acid differences in HSF1a while three in HSF1d among two subspecies. The identity of HSF1a and HSF1d are 97%. A 14-amino acid difference was found between the functional domains of A-HSF1a and A-HSF1d (Supplementary Figure 1).

**TABLE 3:** Differential amino acids of HSF1 between the Pacific oyster and Fujian Oyster.

	*C. gigas gigas*	*C. gigas angulata*	Position
HSF1a	D	G	23
	G	S	34
	G	V	72
	A	G	200
	G	D	321
	S	P	339
	T	A	349
	S	C	355
HSF1d	G	A	200
	P	S	339
	L	M	385

**FIGURE 3:**
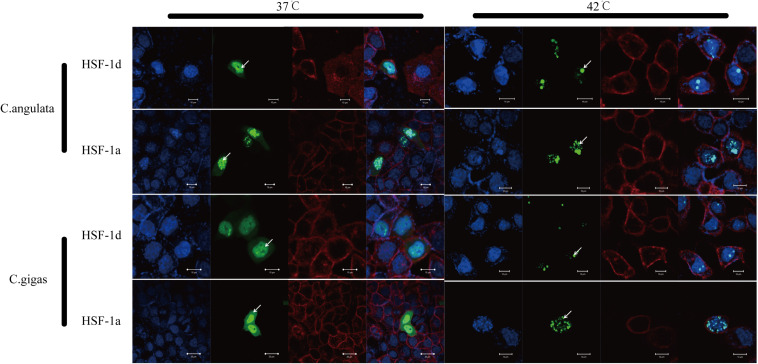
Subcellular localization results for HSF1a and HSF1d in two congener species. HeLa cells were used in the experiment under 37°C (no heat stress) and 42°C (heat shock) treatments. There are four photos for each condition and gene; they are, from left to right: (blue) Hoechst 33342 stained cell nuclei, (green) green fluorescence of genes, (red) Alexa Fluor 633 stained cell membrane, and the fourth is a merged view of the three.

**FIGURE 4:**
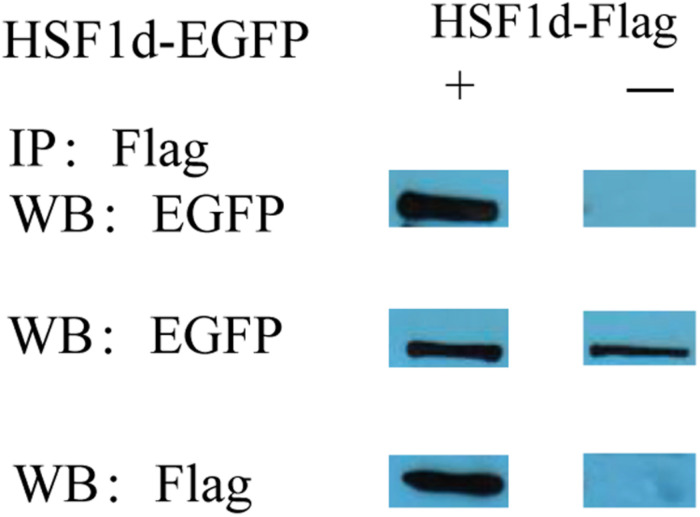
Co-immunoprecipitation showing that HSF1d as a dimer. HSF1d proteins carrying Flag or EGFP tags were co-overexpressed in HEK293T cells, and the interactions were determined by co-immunoprecipitation assays using M2 anti-FLAG antibody. The top boxes are anti-EGFP pulldown. The middle boxes are western blotting with anti- EGFP antibody. The bottom boxes are western blotting with the anti-FLAG antibody.

The subcellular localization results showed that two subtypes (HSF1a and HSF1d) were evenly distributed throughout the cell under no thermal stress, but gathered in the nucleus in a dotted distribution under thermal stress (Figure 3). The co-immunoprecipitation result showed that the EGFP tagged protein can be detected in the samples which contain the FLAG tagged protein and then incubated with anti-FLAG M2 magnetic beads (Figure 4). The EGFP and FLAG tagged proteins were both of HSF1d, which shows that HSF1d can dimerize in cells.

### *HSP* Sequences

The HSPs’ CDS sequences were similar between *C. gigas gigas* and *C. gigas angulata* (Supplementary Figures 2–6). The identities of candidate genes (identity 89–99%, amino acid score 1,186–1,895) are listed in Supplementary Table 3. The functional domains of the corresponding genes in the two subspecies were the same, while their promoter regions had several differences. The potential HSEs of the candidate genes are displayed in Supplementary Figure 7. Statistics regarding the potential HSEs are listed in Table 4.

**TABLE 4:** The statistics of differences in the promoter of the heat shock proteins which can be possible binding sites in two subspecies.

Gene ID	Annotated	Total potential HSE* (number)		Differential potential HSE between *C. gigas gigas* and *C. gigas angulata* (number)
		
		*C. gigas gigas*	*C. gigas angulata*	
*CGI_10006977*	HSP40	18	17	1
*CGI_10002375*	HSP60	40	37	5
*CGI_10008834*	HSP70	24	22	2
*CGI_10002594*	HSP70	48	47	9

“*” potential HSE represent sequences “nGAA” or “nTTCn.”

### Regulatory Relationship Between HSF1 and HSPs

**FIGURE 5:**
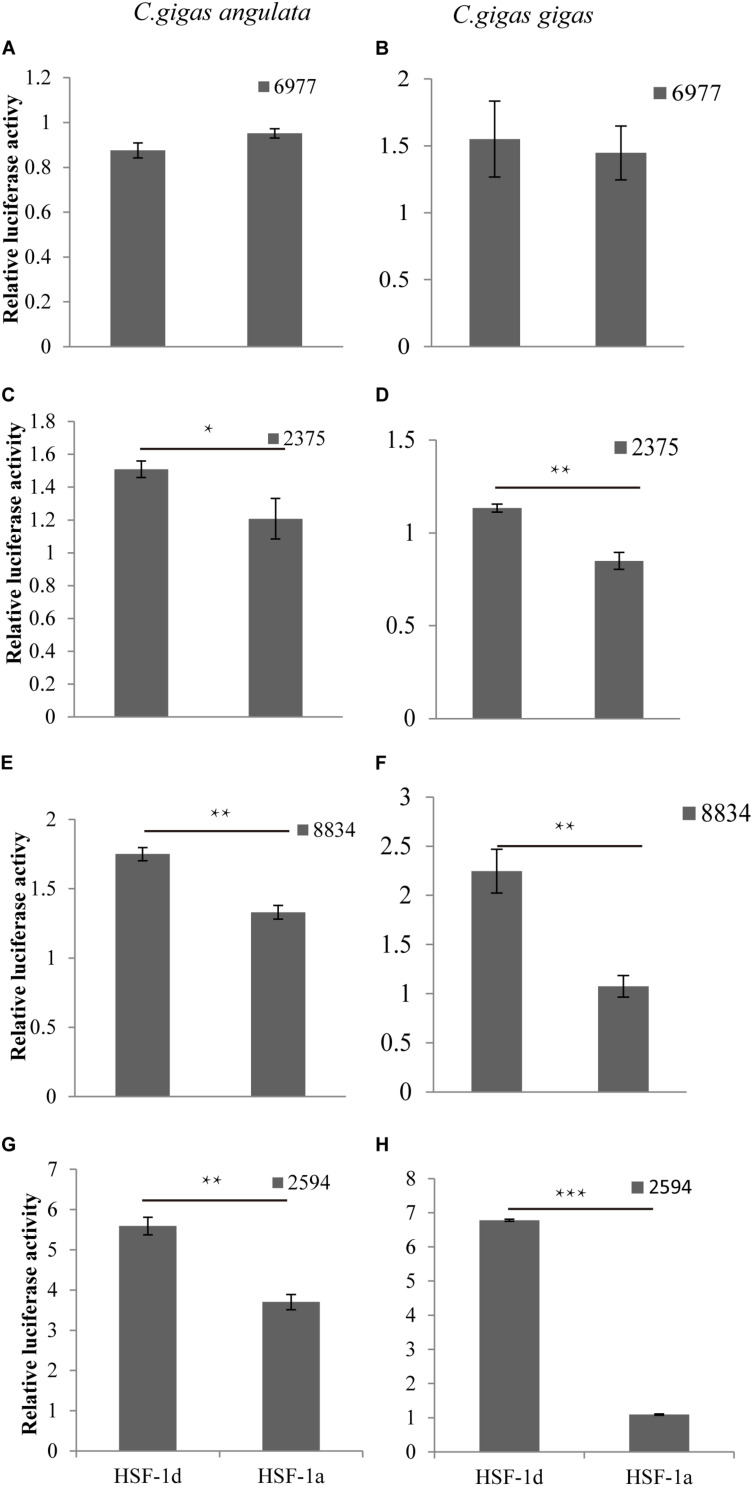
The regulatory of subtypes and the *HSP*s in *C. gigas gigas* and *C. gigas angulata* by dual Luciferase assay. **(A,B)** Relative luciferase activity of HSF1 isoforms with 6977(*CGI_10006977*) active in the Pacific and Fujian oysters, respectively. **(C,D)** Relative luciferase activity of HSF1 isoforms with 2375(*CGI_10002375*) active in Pacific and Fujian oysters, respectively. **(E,F)** Relative luciferase activity of HSF1 isoforms with 8834(*CGI_10008834*) active in the Pacific and Fujian oysters, respectively. **(G,H)** Relative luciferase activity of HSF1 isoforms with 2594(*CGI_10002594*) active in the Pacific and Fujian oyster, respectively. The horizontal axis represents HSF1d and HSF1a, and the vertical axis represents the relative luciferase activity of the target plasmid (HSF1d or HSF1a). The left column is under HSF1d activation while the right one is under HSF1a activation. Error bars denote the standard error of the mean. *0.01 < *p* < 0.05, **0.001 < *p* < 0.01, ***p < 0.001.

The regulatory relationship between HSF1 and HSPs in the two congener species is shown in Figure 5. The four histograms on the left represent regulation in the Fujian oyster while those on the right represent that in the Pacific oyster. The results show that the level of activation regulation in *CGI_10006977* (Figures 5A,B) was relatively low and it was not significantly different between the two subtypes (*P* > 0.05). The other three genes—*CGI_10002375* (Figures 5C,D), *CGI_10008834* (Figures 5E,F), and *CGI_10002594* (Figures 5G,H)—on the other hand, could be significantly activated by both subtypes (*P* < 0.05); the HSF1d isoform had a strong activating ability in both oysters.

## Discussion

### HSF1 Isoforms Suggest the Potential for Differential Regulation

Sequence variation among HSF1 isoforms revealed a differential functional domain. The expression levels of other subtypes were extremely low compared to those of HSF1a and HSF1d, which may suggest that the two isoforms are response genes for thermal stress. In this study, two HSF1 isoforms, A-HSF1a and A-HSF1d, were cloned from thermal stress-treated Fujian oyster. The corresponding variable splicing protein coding regions are almost the same in the two subspecies, and the 14-amino acid deletion previously found in the Pacific oyster (Kawabe and Yokoyama, 2011) also exists in the Fujian oyster. The functional domain of HSF1 is divided into binding and activation regions. Different parts of the domain have different functions, which may affect how they regulate downstream genes (Xie et al., 2003; Wang et al., 2018).

HSF1 isoforms displayed different expression patterns under thermal stress (*P* > 0.05). Total HSF1 expression peaked at 36°C in the Pacific oyster and 29°C for the Fujian oyster. The expression level is higher in the Pacific oyster compared with the Fujian oyster among different treatments. Some researcher reported that the Fujian oyster living a higher average temperature than the Pacific oyster (Li et al., 2017). It might consist with our result that the Fujian oyster with an easy trigger but small response heat shock defense system. HSF1d had the highest expression in both subspecies, while HSF1a showed a lower expression. In addition, the two subtypes responded differently to the various temperature stimuli. A previous study on mice showed that different subtypes of HSF1 may be synergistically regulated, meaning that different proportions of subtypes would contribute to different levels of HSP genes by transcriptional regulation (Neueder et al., 2014).

Studies have reported that the specialization of multiple HSF subtypes can increase the transcription rate of heat shock genes (Morimoto, 1998), which can reflect the proportion of different subtypes expressed in the two subspecies when coping with different temperatures and pressures. To some extent, the present study suggests that different proportions of HSF1 subtype combinations play an important regulatory role in regulating HSPs in oysters.

These subtypes exist in oysters, but there are different combinations of heat shocks, and this complicates the question of whether these subtypes play a role. This study judged whether the subtypes responded to thermal stimulus by whether its localization to thermal stimulus changed in HeLa cells. The results of this part of the experiment show that the subtypes in both subspecies can respond to heat shock stimuli and gather in the nucleus in a point-like distribution. Some scholars have reported that HSF1 is regulated by aggregating into a trimer in the nucleus and binding to the binding site of the promoter region (Ortner et al., 2015; Gomezpastor et al., 2018). It can be speculated that when cells receive stress signals, proteins accumulate in the nucleus and play a role in regulating the expression of heat shock response genes, thereby protecting the body from heat shock damage. The results of this study suggest that HSF1 regulation in oysters is similar to that of model animals by combining specific sites in the nucleus.

The results show that HSF1 can form aggregates and perform regulatory functions. As previously described, the monomerization- and trimerization processes can account for the entire process of heat shock, and the polymer is what plays the regulatory role (Morimoto, 1993). The combination experiment in this study proves that HSF1 in oysters can be combined, providing the possibility for it to be regulated.

### Heat Shock Protein Genes in Oysters

In this study, analyzing the coding region of HSPs showed that the corresponding genes of the two subspecies are conserved, while the promoter region showed a high variation in HSP genes in both subspecies. The promoter region is important for regulation because of its sequence specificity. Previous studies showed that the two congener species responded to heat shock by expressing the *HSP* genes differentially (Ghaffari et al., 2019; Li et al., 2019). A previous study showed that *HSP*s are regulated by HSF1 (Wu, 1995). The HSEs were important in the regulatory relationship for HSF1 and *HSP*s. Our result showed that HSEs in the promoter regions varied significantly between the two subspecies, and this has an important influence on regulation. Studies of gene binding site mutations in many other fields indicate that mutations in potential binding sites may interfere with that site’s original regulatory function (Lam et al., 1989; Ono et al., 2001). Some scholars found that polymorphisms in the promoter region of an oyster serine protease inhibitor gene are associated with parasite resistance (He et al., 2012).

The copepod transcriptome data revealed that *HSP* homologous genes responded differently to thermal stimuli in different populations, and the HSP70 family is the clearest example of this (Schoville et al., 2012). This means that HSP genes are differentially expressed in response to heat shock in different populations and even subspecies. The gene *CGI_10006977* (*HSP40*) has the fewest number of potential HSEs and differential sites in the two subspecies, and *CGI_10002594* (*HSP70*) has the most. A previous study showed that *CGI_10006977* and *CGI_10002594* were both upregulated in response to heat shock, and that the expression levels of *CGI_10006977* are very similar between the two subspecies while those of *CGI_10002594* are significantly different (*p* < 0.01) (Li et al., 2019). In addition, a study showed that under the same heat stress as the experiment design in this paper, the *CGI_10006977* gene is not significantly different under Two-way ANOVA analysis with temperature and species factors, while the *CGI_10008834* and *CGI_10002387* genes are significantly expressed (Ghaffari et al., 2019). This suggests that differences exist in how HSP expression is regulated among subspecies with different thermal tolerances.

### HSF1 May Contribute to HSP Gene Expression Levels Under Thermal Stress

The regulation of HSF1 isoforms and *HSP*s showed that the HSF1d has stronger induction ability than HSF1a in both the Pacific and Fujian oysters. The induction ability of HSF1d in the Pacific oyster was stronger than that of the Fujian oyster. For HSF1a, the induction ability of the Fujian oyster is higher than that of the Pacific oyster. A previous study indicated that Pacific oysters respond to stress by inducing *HSP* expression, while Fujian oysters may have a higher threshold for heat-induced stress (Ghaffari et al., 2019). Studies showed that the HSF1a is a constitutively expressed isoform, while HSF1d is inducible under air exposure and/or hypoxia (Kawabe and Yokoyama, 2011). The expression pattern results in Figure 1 showed that HSF1d was more highly expressed than HSF1a under the same thermal stress conditions. HSF1a changed a lot in response to different temperature stresses. A previous study showed that HSF1a and HSF1d have different activities when *HSP* is activated (Liu et al., 2019). In Figure 5, the regulation of *CGI_10006977* showed no significant (*p* > 0.05) activity between HSF1a and HSF1d in either subspecies. The result of *CGI_10002594* activated by HSF1a and HSF1d showed a significant (*p* < 0.01) difference in the two subspecies. In addition, the *CGI_10002594* sequence had the largest difference in the promoter region and was also activated the most by HSF1 in the luciferase double reporter experiment. HSE studies on Drosophila HSP70 have shown that the size, location, and sequence of HSE affects the organism’s response to heat shock, and it may be a combination of changes that gives it versatility in regulating stress (Tian et al., 2010). The region in the gene in which the two subspecies differ may have a critical effect on regulation. This study also shows that the existence of multiple types of HSE may also affect gene regulation. We argue that HSF1d is an inducible expression-type protein and HSF1a is a basic expression protein; this may explain the phenomenon that a larger HSP induced expression in the Pacific oyster while Fujian oysters have a higher basic expression.

## Conclusion

In this study, the HSF1 isoforms HSF1a and HSF1d were studied by examining their expression levels, localization, and self-interactions. These isoforms respond in different ways to heat shock and different temperature stimuli. The amino acids encoded by the HSP genes of *C. gigas angulata and C. gigas gigas* are conserved while the promoter regions vary. In addition, HSF1d has a stronger gene activation effect in the two subspecies and HSF1a has a stronger activation effect in the Fujian oyster. Most of the induced genes in the subspecies appear to be induced by HSF1d more than by HSF1a. A gene with a large sequence difference (*CGI_10002594*) yielded the most activation thresholds, indicating that the difference in the sequence between the two subspecies may affect the regulatory effect. These findings will be of interest to future studies aiming to understand the mechanisms underlying thermal tolerance.

## Data Availability Statement

The sequences of A-HSF1a, A-HSF-1d, and HSPs used in this study can be found in the GeneBank (https://www.ncbi.nlm.nih.gov/genbank/, accession numbers MT737786 to MT737796).

## Ethics Statement

The animal study was reviewed and approved by the Institute of Oceanology, Chinese Academy of Sciences.

## Author Contributions

LL and GZ conceived the project. YL, LL, and GZ participated in the design of the study and discussed the results. YL conducted the RNA extraction and HSF1 and HSPs’ cloning and wrote the manuscript. YL, HQi, and HQue carried out the bioinformatics analyses. YL, HQi, HQue, and LL polished the manuscript. WW collected the experimental materials. All authors have read and approved the final manuscript.

## Conflict of Interest

The authors declare that the research was conducted in the absence of any commercial or financial relationships that could be construed as a potential conflict of interest.

## Footnotes

1 http://www.mbio.ncsu.edu/BioEdit/

2 https://www.ebi.ac.uk/Tools/msa/clustalo/

3 http://smart.embl-heidelberg.de/

4 https://www.ncbi.nlm.nih.gov/Structure/cdd/wrpsb.cgi

5 http://www.detaibio.com/sms2/protein_mw.html

6 https://blast.ncbi.nlm.nih.gov/Blast.cgi?PAGE_TYPE=BlastSearch&PROG_DEF=blastn&BLAST_PROG_DEF=blastn&BLAST_SPEC=GlobalAln&LINK_LOC=BlastHomeLink

## Supplementary Material

Supplementary Figure 1 Differential coding sequences of HSF1a and HSF1d.

Supplementary Figure 2 Information of the heat shock protein *CGI_10006977*.

Supplementary Figure 3 Information of the heat shock protein *CGI_10008834*.

Supplementary Figure 4 Information of the heat shock protein *CGI_10004164*.

Supplementary Figure 5 Information of the heat shock protein *CGI_10017582*.

Supplementary Figure 6 Information of the heat shock protein *CGI_10009495*.

Supplementary Figure 7 Potential heat shock elements in four heat shock proteins’ promoter.

Supplementary Table 1 Primers used for coding sequences amplification.

Supplementary Table 2 Gene expression levels of HSF1 isoforms in the two subspecies.

Supplementary Table 3 Statistic of the sequences’ identity for HSP genes in the Pacific oyster and Fujian oyster.
