# Supplementary material for: Regulation Between HSF1 Isoforms and HSPs Contributes to the Variation in Thermal Tolerance Between Two Oyster Congeners
Source: Front Genet. 2020 Oct 27;11:581725. doi: 10.3389/fgene.2020.581725 (PMC7652795; doi:10.3389/fgene.2020.581725)
Supplement: Supplementary Figure 1 — Differential coding sequences of HSF1a and HSF1d. [file Data_Sheet_1.docx]

Supplementary Material

# Supplementary Figures and Tables

## Supplementary Figures


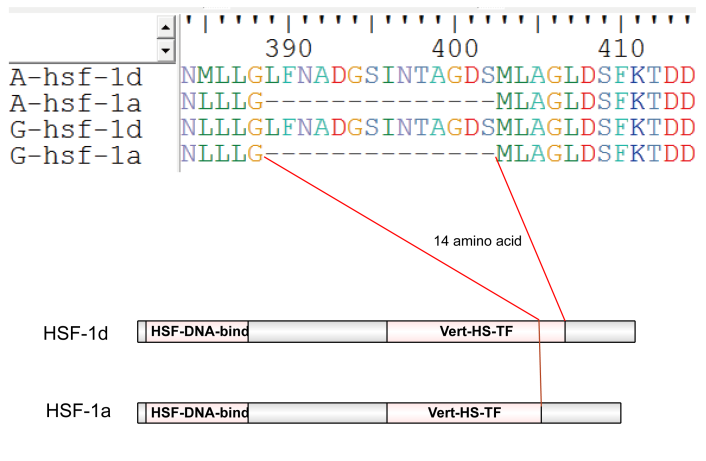


**Supplementary Figure 1.** Differential coding sequences of HSF1a and HSF1d. The “A-hsf-1d,” “A-hsf-1a,” “G-hsf-1d,” and “G-hsf-1a” represent HSF1d in Fujian oyster, HSF1a in Fujian oyster, HSF1d in Pacific oyster, and HSF1a in Pacific oyster, respectively. The upper sequences represent the amino acids of genes, and “390, 400, 410” represent the location of the amino acids. The bottom two columns represent the functional domain of HSF1d and HSF1a, respectively.


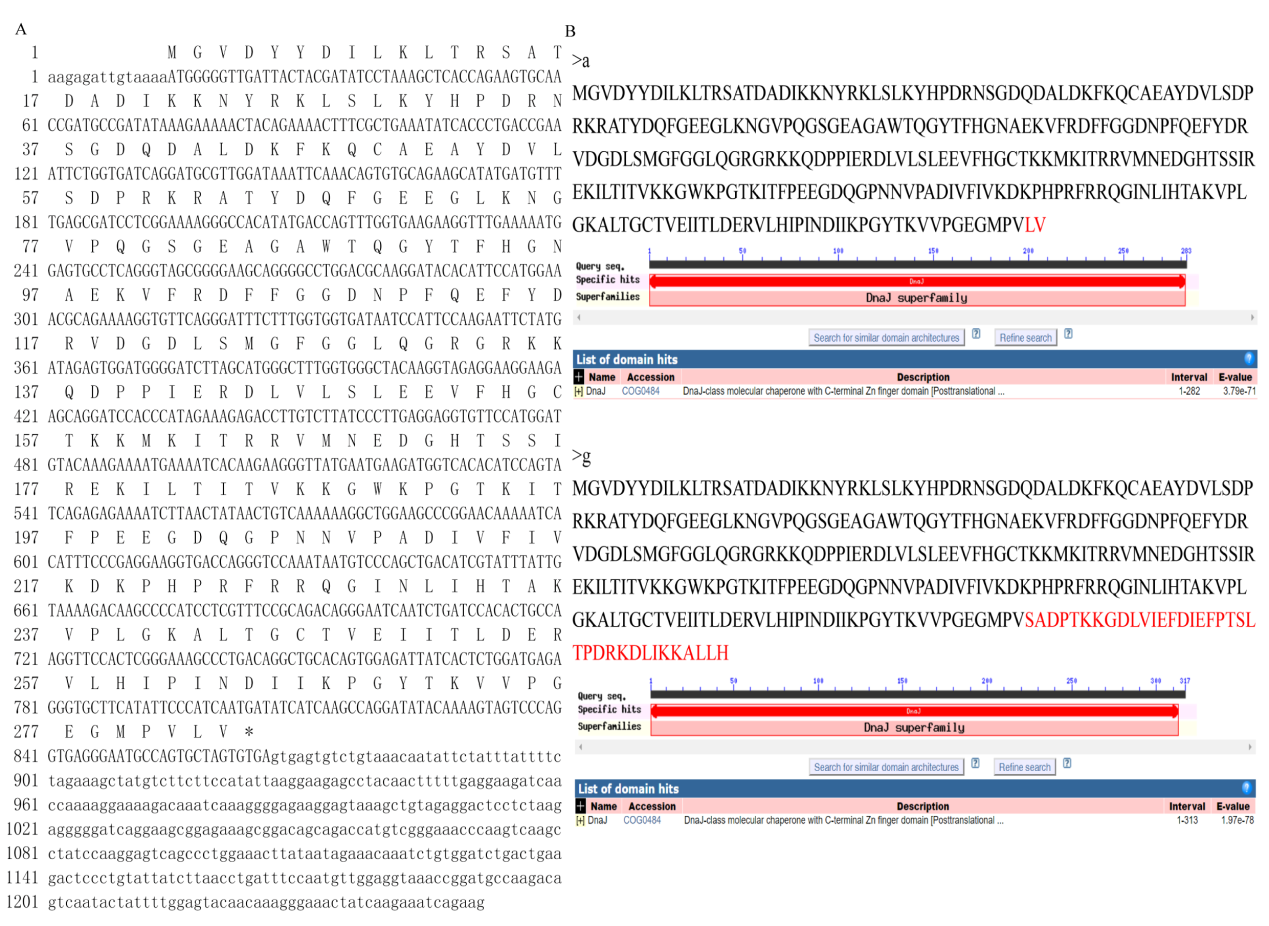


**Supplementary Figure 2.** Information of the heat shock protein *CGI_10006977*.

(A) Sequence information of *CGI_10006977* nucleic acid and protein. The upper row is for amino acids and the bottom is for nucleic acid sequences.

(B) Comparison of encoded proteins and functional domains. "a" stands for *C. gigas angulata*, and "g" stands for *C. gigas gigas*. The different amino acids in two subspecies are marked in red.


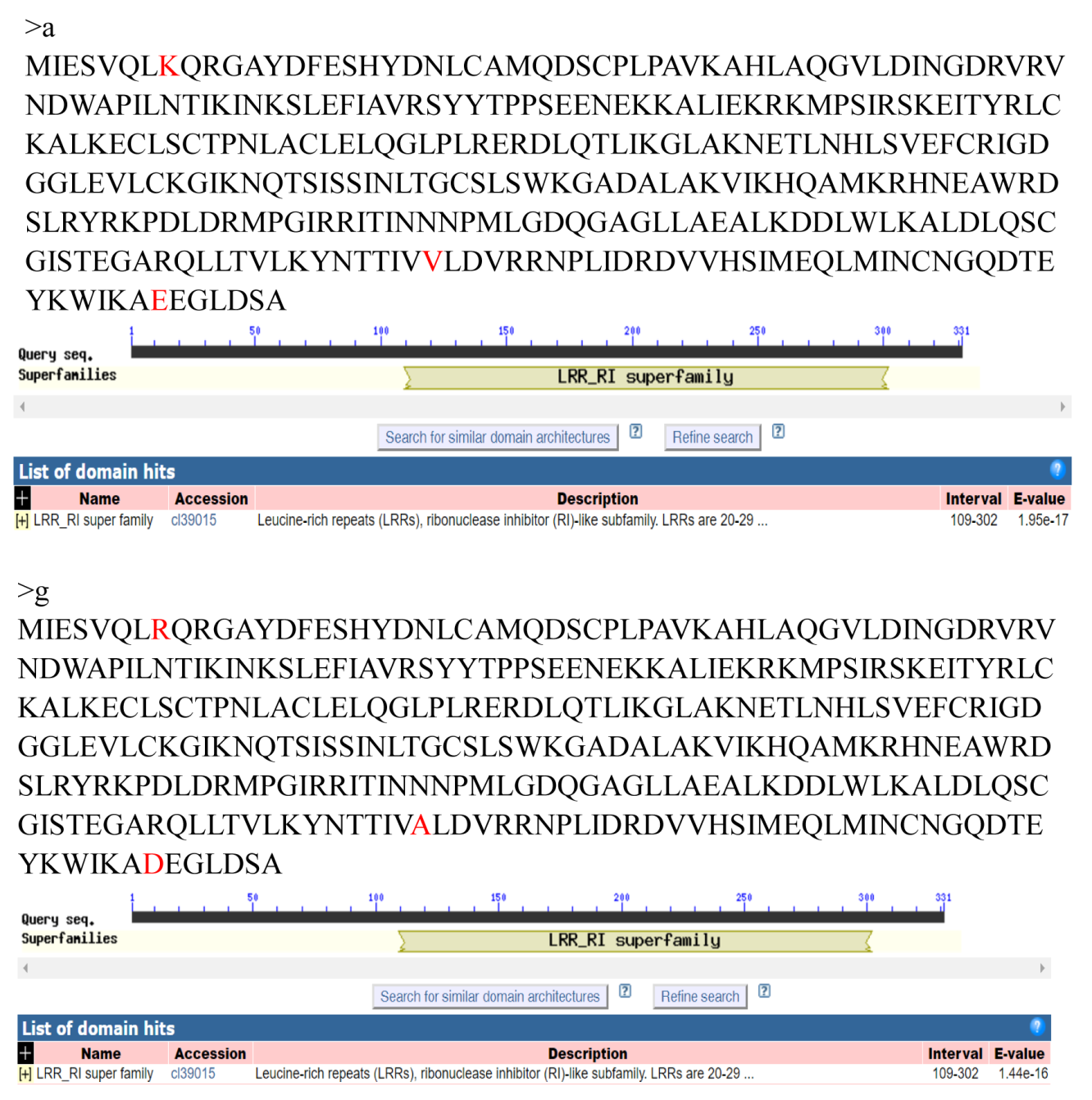


**Supplementary Figure 3.** Information of the heat shock protein *CGI_10008834*.

We compared of encoded proteins and functional domains in two subspecies. "a" stands for *C. gigas angulata*, and "g" stands for *C. gigas gigas*. The different amino acids in two subspecies are marked in red.


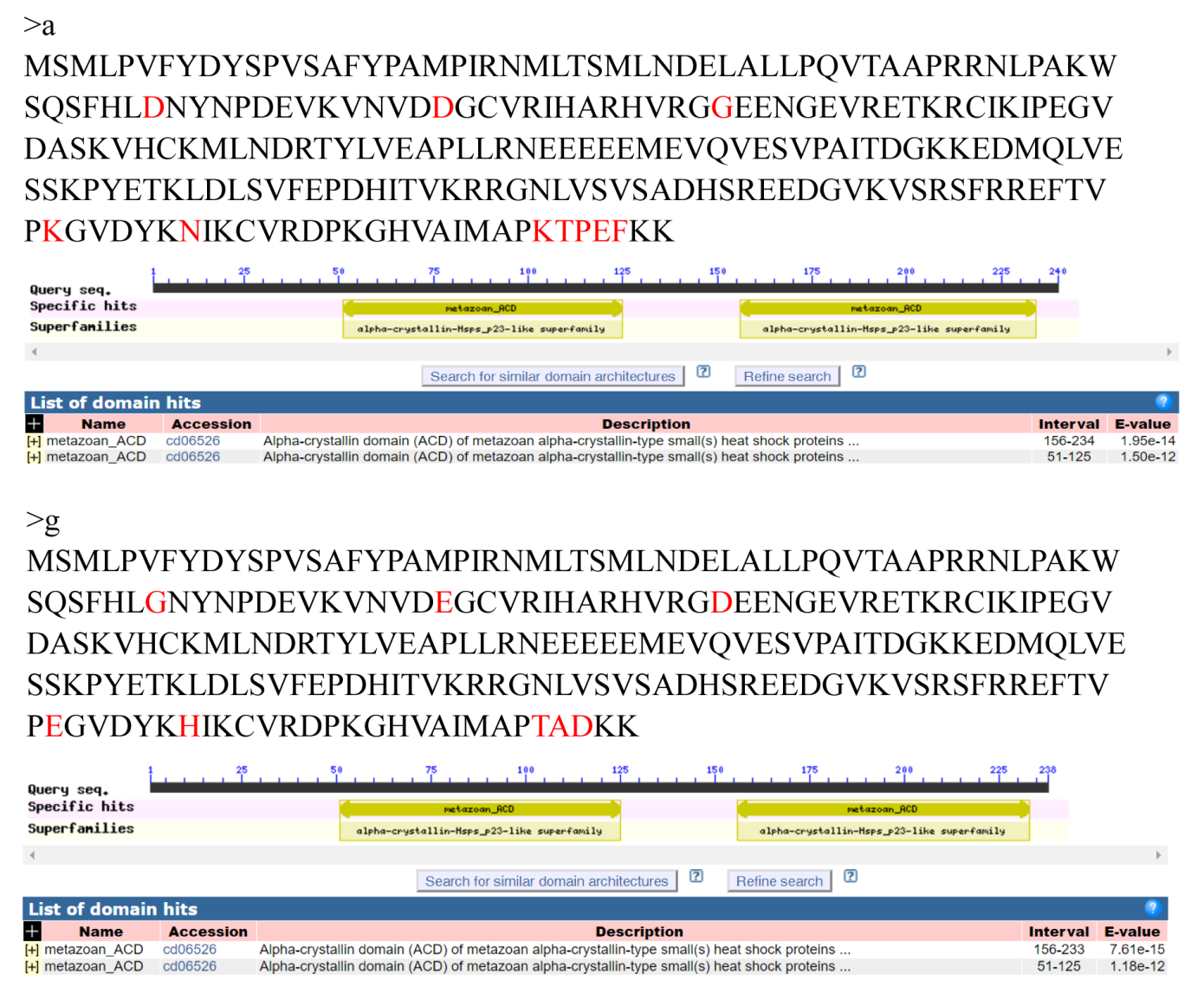


**Supplementary Figure 4.** Information of the heat shock protein *CGI_10004164*.

We compared of encoded proteins and functional domains in two subspecies. "a" stands for *C. gigas angulata*, and "g" stands for *C. gigas gigas*. The different amino acids in two subspecies are marked in red.


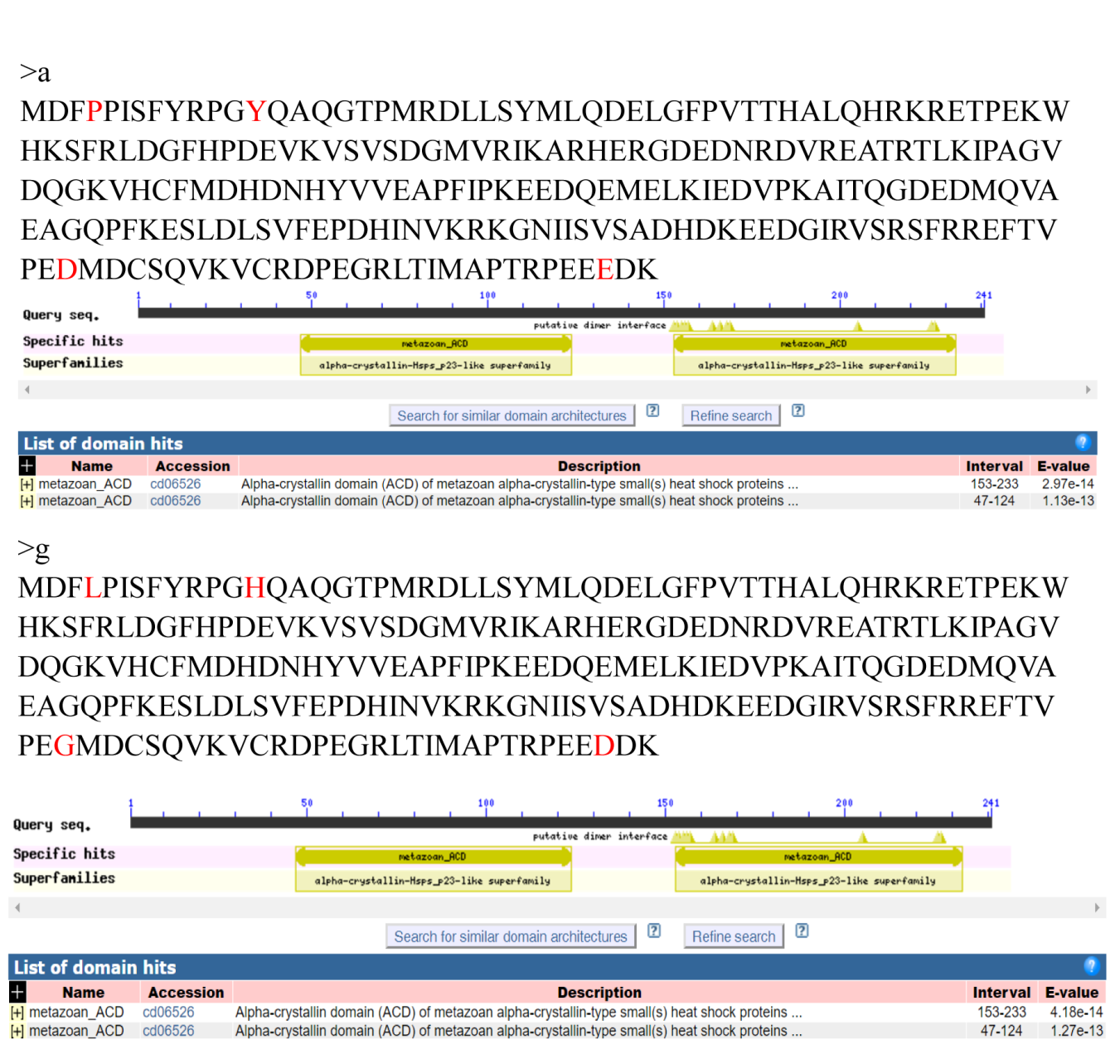


**Supplementary Figure 5.** Information of the heat shock protein *CGI_10017582*.

We compared of encoded proteins and functional domains in two subspecies. "a" stands for *C. gigas angulata*, and "g" stands for *C. gigas gigas*. The different amino acids in two subspecies are marked in red.


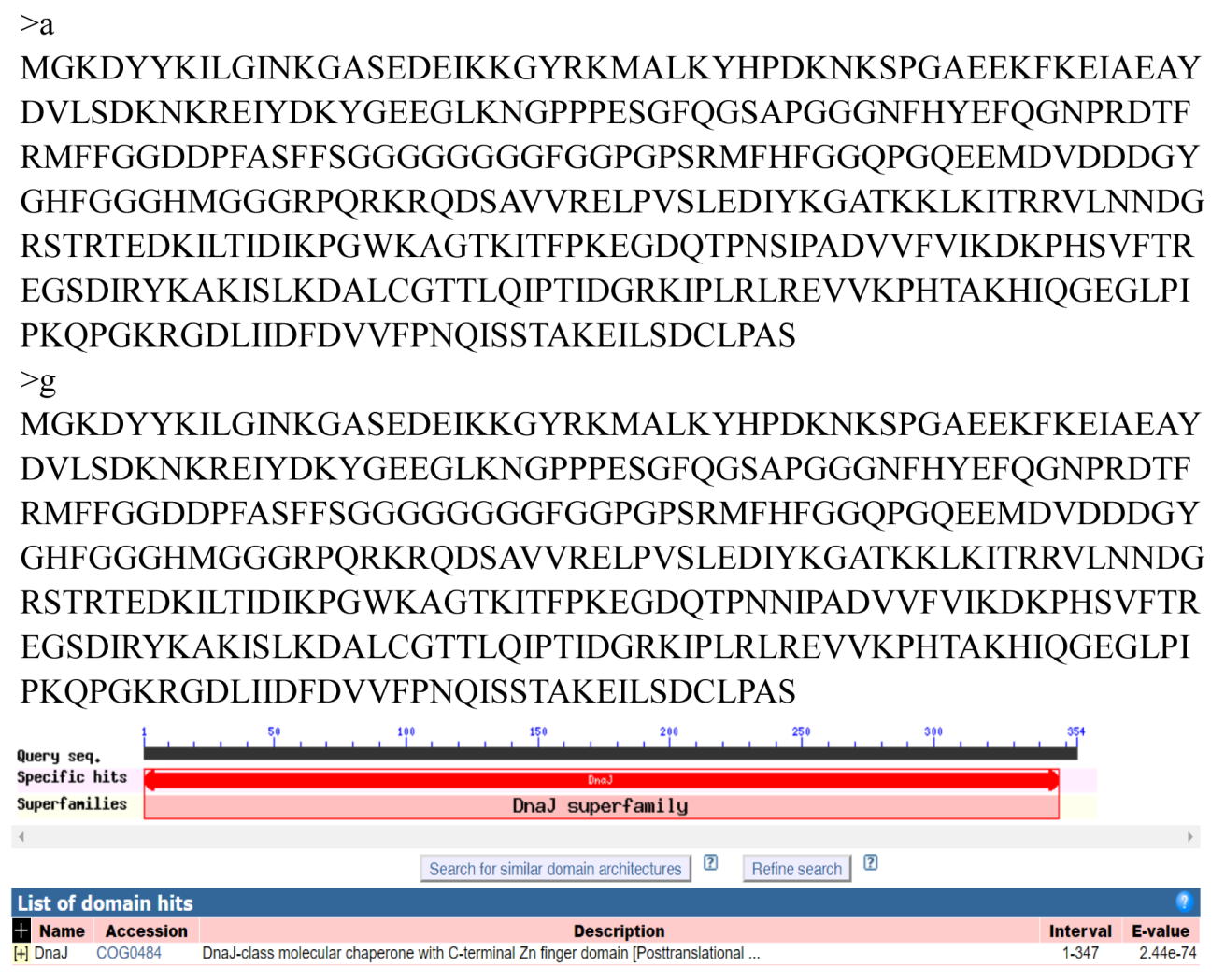


**Supplementary Figure 6.** Information of the heat shock protein *CGI_10009495*.

We compared of encoded proteins and functional domains in two subspecies. "a" stands for *C. gigas angulata*, and "g" stands for *C. gigas gigas*. Their amino acids in two subspecies are same.


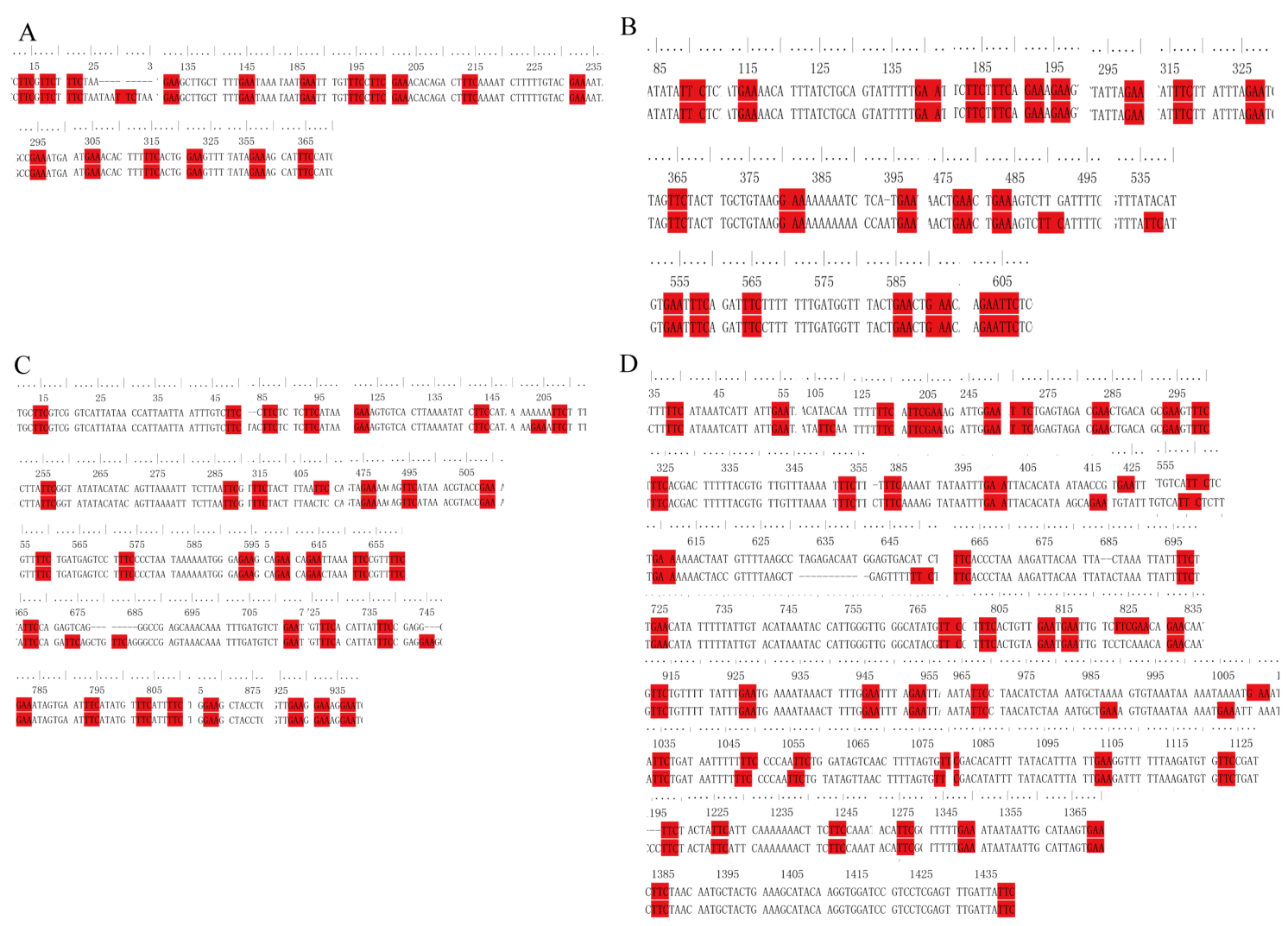


**Supplementary Figure 7.** Potential heat shock elements in four heat shock proteins’ promoter.

(A), (B), (C), (D) are represent potential HSEs in the promoter of *CGI_10006977*, *CGI_10008834, CGI_10002375*, and *CGI_10002594*, respectively. The letters with highlight are “TTC” or “GGA”, and the numbers on the top of sequences represent the location of nucleotides.

## Supplementary Table

**Supplementary Table 1.** Primers used for coding sequences amplification.

| Name | Sequences |
| --- | --- |
| HSF-1F | ATGGGTTCAAACCCTGTACCAGCG |
| HSF-1R | CAGGTCGTCTGCGGAGATCTGG |
| 10008834CDS-F | GACAGCGGGGGGCATACG |
| 10008834CDS -R | CCCTGGTCTCCAAGCATCGG |
| 10004164 CDS-F | GTCAGAATTCACGCTCGTCATG |
| 10004164 CDS-R | GAATGAACGAGACACTTTGACCC |
| 10006977 CDS-F | GAGTGCCTCAGGGTAGCGG |
| 10006977 CDS-R | AGGTCCTTTCTGTCGGGGG |
| HSF-5’-R | CCAGCGTTTTTGACCAAGTTA |
| HSF-3’-F | CACACCCCTACCCATCCAG |
| 10008834-R1 | GACGGCAGGTAAGGGGCA |
| 10008834-R2 | GGCAGGTAAGGGGCAGGAA |
| 10008834-R3 | ACTCTCAAAGTCGTATGCCCCCC |
| 10008834-F3 | GGGACAGCCTCCGTTACAGAAAA |
| 10008834-F2 | CGGAGGATTGGAGGTGCTTTGTAA |
| 10008834-F1 | TGTAGAATCGGGGACGGAGGA |
| 10004164-R3 | GCGGACTTCGCCGTTCTCTT |
| 10004164-R2 | GGTGCGGTCATTCAGCATCTTA |
| 10004164-R1 | GCCTCAACAAGGTAGGTGCGGTCA |
| 10004164-F3 | TGGGGTCAAAGTGTCTCGTTCA |
| 10004164-F2 | GAACTTGGTGTCCGTGTCTGCTG |
| 10004164-F1 | TTACGGACGGCAAGAAAGAAGAC |
| 10006977-R3 | GCTTCCCCGCTACCCTGAG |
| 10006977-R2 | GACAAGGTCTCTTTCTATGGGTGG |
| 10006977-R1 | GTGTGACCATCTTCATTCATAACCCT |
| 10006977-F3 | CCAGGTGAGGGAATGCCAGTG |
| 10006977F2 | CACTGCCAAGGTTCCACTCG |
| 10006977F1 | CACACTGCCAAGGTTCCACTCG |
| 10017582-R3 | GGGTTTCTCTCTTTCTGTGCTGC |
| 10017582-R2 | CCATCCCATCACTCACCGAAA |
| 10017582-R1 | CCTTACCATCCCATCACTCACC |
| 10017582-F3 | GAGGAGGACGGGATAAGGGTG |
| 10017582-F2 | GGCAACATCATTTCCGTAAGCG |
| 10017582-F1 | CAAACGCAAGGGCAACATCA |
| 10009495F3 | TGAAACCCCACACAGCCAAGCA |
| 10009495F2 | GATTCCCCTGCGACTACGG |
| 10009495F1 | GAAAGATTCCCCTGCGACTACGG |
| 10009495R3 | ACGGGTCGTCTCCTCCAAAG |
| 10009495R2 | GCAAACGGGTCGTCTCCTCCAA |
| 10009495R1 | ATTTCTTCCTGACCTGGTTGCCC |

**Supplementary Table 2.** Gene expression levels of HSF1 isoforms in the two subspecies.

|  | *C. gigas gigas* | | | | | *C. gigas angulata* | | | | |
| --- | --- | --- | --- | --- | --- | --- | --- | --- | --- | --- |
|  | 22°C | 29°C | 36°C | 40°C | 43°C | 22°C | 29°C | 36°C | 40°C | 43°C |
| HSF1-total | 1.016 | 1.149 | 1.452 | 0.977 | 1.111 | 0.586 | 0.924 | 0.823 | 0.567 | 0.494 |
| **HSF1a** | 0.105 | 0.136 | 0.282 | 0.220 | 0.169 | 0.053 | 0.045 | 0.057 | 0.090 | 0.052 |
| HSF1b | 0.027 | 0.042 | 0.040 | 0.031 | 0.033 | 0.006 | 0.007 | 0.014 | 0.003 | 0.012 |
| HSF1c | 0.001 | 0.005 | 0.001 | 0.001 | 0.002 | 0.000 | 0.000 | 0.001 | 0.001 | 0.000 |
| **HSF1d** | 0.395 | 0.418 | 0.362 | 0.255 | 0.312 | 0.214 | 0.237 | 0.262 | 0.200 | 0.194 |
| HSF1e | 0.001 | 0.003 | 0.000 | 0.000 | 0.000 | 0.000 | 0.000 | 0.000 | 0.000 | 0.000 |
| HSF1f | 0.209 | 0.143 | 0.094 | 0.061 | 0.087 | 0.062 | 0.032 | 0.081 | 0.026 | 0.033 |
| HSF1g | 0.018 | 0.045 | 0.006 | 0.005 | 0.005 | 0.005 | 0.024 | 0.003 | 0.004 | 0.005 |
| HSF1h | 0.010 | 0.043 | 0.004 | 0.003 | 0.003 | 0.004 | 0.008 | 0.003 | 0.000 | 0.000 |

**Supplementary Table 3.** Statistic of the sequences’ identity for HSP genes in the Pacific oyster and Fujian oyster.

| Gene | identity | score |
| --- | --- | --- |
| CGI_10006977 | 89% | 1460 |
| CGI_10004164 | 96% | 1186 |
| CGI_10008834 | 99% | 1714 |
| CGI_10017582 | 98% | 1259 |
| CGI_10009495 | 99% | 1895 |
